# The Requirements and Development Potential of Interdisciplinary Digital Health Data Exchange in Mobile Nursing and Care Settings in German-Speaking Countries: Delphi Study

**DOI:** 10.2196/78193

**Published:** 2025-08-13

**Authors:** Verena Kollmann, Nathalie Traugott, Susanne Hensely-Schinkinger, Doris Zeidler, Elisabeth Haslinger-Baumann

**Affiliations:** 1 Center for Applied Nursing Research FH Campus Wien Vienna Austria; 2 Research Center Digital Health and Care FH Campus Wien Vienna Austria; 3 Vice-Rector for Research and Development FH Campus Wien Vienna Austria

**Keywords:** electronic health records, health information exchange, nursing informatics, home care services, nursing, Delphi study, interprofessional relations, Germany, Austria, Switzerland

## Abstract

**Background:**

The integration of electronic health records (EHRs) in mobile care presents complex challenges in German-speaking countries (DACHL region: Germany, Austria, Switzerland, and Liechtenstein). While digitalization efforts are advancing, fragmented infrastructure, unclear access rights, and inconsistent implementation strategies hinder interdisciplinary data exchange, particularly in mobile nursing and care settings.

**Objective:**

This study aimed to explore expert perspectives on the requirements and challenges for cross-sectoral integration of EHRs in mobile care within the DACHL region; essential system functions and support structures; and the expected impact of digital data exchange on care quality, workload, and collaboration.

**Methods:**

A modified Delphi approach was used, comprising 2 phases. In phase 1, qualitative expert interviews were conducted between January and April 2024, using a semistructured interview guide. Interviews were analyzed using qualitative content analysis to derive key findings and thematic groups. These results informed the design of a structured online survey. In phase 2, 2 Delphi rounds were carried out (round 1: April/May 2024; round 2: July/August 2024). The first round included 159 items rated on a 4-point Likert scale and a ranking task of EHR functions. In the second round, only items without prior consensus and the top-ranked functions were reassessed. Consensus was defined as ≥75% agreement or disagreement among participants.

**Results:**

Nineteen experts participated in qualitative interviews, 18 in the first survey round, and 15 in the second round (retention rate: 79%). Consensus was reached for 141 of the 159 survey items (88.7%). Experts emphasized the importance of open, interoperable systems; standardized terminologies; and agile development tailored to mobile care. High agreement was identified for the relevance of key EHR functions, with diagnoses, medication lists, assessments, and medical history showing the highest ranking scores in round 2 (32, 27, 19, and 18 points of 48 possible points, respectively). Usability and differentiated support structures were considered essential for successful implementation. Cross-border data exchange, telemonitoring, and artificial intelligence integration were seen as valuable, while the topic of access rights, particularly for assistant roles, provoked the most disagreement, indicating a need for further clarification in policy and practice. All panelists (17/17, 100%) endorsed open, interoperable systems and rigorous usability testing, whereas only 23% (3/13) agreed on granting full data access rights to assistant roles. Overall, experts predicted that interoperable EHRs would improve patient safety (15/17, 88%), reduce duplicate documentation (16/17, 94%), and enhance interdisciplinary collaboration (16/16, 100%).

**Conclusions:**

Experts in the DACHL region broadly agreed on the functional and structural key requirements for effective digital data exchange in mobile care. Interdisciplinary EHR implementation must emphasize interoperability, context-sensitive access policies, and usability. The findings provide a foundation for policy development, system design, and future research, contributing to safe and efficient digital care delivery across sectors and borders.

## Introduction

### Background

The digitization of the health care system is a major opportunity for the improvement of the quality and efficiency of health care services [1]. Recent bibliometric evidence confirms a steady rise in health information exchange research, extending to nursing use cases [2]. Driven by the COVID-19 pandemic, the European Health Data Space (EHDS), approved by the European Parliament in April 2024, aims to establish a comprehensive ecosystem for health data exchange across European Union (EU) member states. This initiative addresses the current challenges in accessing and sharing health data electronically, particularly when patients move within or between EU countries [3]. However, this digital transformation also brings substantial risks and challenges, especially regarding institutional trust, legal compatibility, and technical operability across borders [4]. The national legislation and political interests of the individual countries make it difficult to achieve a standard and seamless transfer of health data. One reason for this is that there is no standardized nursing terminology, such as Systematized Nomenclature of Medicine–Clinical Terms (SNOMED CT); Logical Observation Identifiers, Names, and Codes (LOINC); and Clinical Care Classification (CCC), across borders. Moreover, there are interoperability issues throughout the different platforms.

German-speaking countries (Germany, Austria, Switzerland, and Liechtenstein), which are collectively referred to as the DACHL region, seem to be geographically, economically, and culturally interconnected, but digitalization and data exchange have progressed differently in the various countries. Germany implemented an electronic patient record (“elektronische Patientenakte” [ePA]) in 2021. Until now, participation in the ePA was voluntary and had to be applied for [5]. Due to the low participation of the population, the system was changed to an opt-out solution in April 2025. This development is primarily due to the slow implementation of standardized telematics infrastructure and ongoing concerns about data protection, both of which have contributed to a general lack of trust in digital health systems [5,6].

The Elektronische Gesundheitsakte (ELGA) has been the Austrian solution for the electronic health record (EHR) since 2015. All persons entitled to health services have access to the ELGA. Individuals can voluntarily decide not to participate and opt out [7]. In Austria, care facilities also have access to the data, unlike in Germany, where only doctors and pharmacies have access [7,8]. Switzerland established the “elektronisches Patientendossier” (EPD) in 2017. However, full implementation in hospitals, nursing homes, and maternity homes has not yet been realized. Registration for the EPD is not mandatory for the public, doctors in private practice, pharmacies, extramural care, and physiotherapists. People are cautious regarding the acceptance and use of the EPD, and it is not widespread [9,10]. Similar to the Austrian system, Liechtenstein also introduced an electronic health dossier (“elektronisches Gesundheitsdossier” [eGD]) in 2023 [11].

The European Commission has made the following statement: “Health data are the blood running through the veins of our health care systems” [12]. Therefore, data should not stand still but flow through the veins of nursing in all settings, regardless of national borders.

The exchange of nursing data faces major challenges due to the limited interoperability of electronic systems. Incompatible technical infrastructure, lack of standardized nursing classifications, and insufficient integration of nursing-specific content hinder effective data sharing [1,13,14]. Moreover, institutional and organizational barriers, such as missing guidelines, lack of digital competence among staff, limited stakeholder engagement, and insufficient incentives for structured documentation, have been repeatedly identified in the literature [15]. Despite these barriers, data exchange is essential to improve patient safety, support evidence-based nursing, and enhance workflow efficiency. Standardized electronic nursing records enable the systematic documentation of nursing interventions and outcomes, thereby fostering continuity of care and enabling secondary data use for research and quality monitoring [13,14]. Nevertheless, empirical studies have emphasized that the mere introduction of digital systems does not automatically improve clinical outcomes; rather, the effectiveness depends on appropriate implementation strategies, contextual adaptation, and evaluation [16]. While some authors have emphasized the transformative potential of digitalization for professionalizing nursing [13], others have highlighted the current lack of conclusive evidence regarding outcome improvements in clinical practice [1]. Moreover, the integration of electronic records into nursing workflows remains uneven and often lacks sufficient technical and educational support [14].

Overall, the literature shows that digital transformation in health and nursing care is not only a technical endeavor but also a complex socioinstitutional process that requires context-sensitive approaches, iterative evaluation, and trust-building mechanisms [4,15,16]. Looking ahead, authors emphasize the need for further standardization and integration of nursing records into broader health systems [13,14]. Digital documentation is seen as key to strengthening nursing autonomy and interprofessional collaboration, with training as a prerequisite [13,14].

Taken together, these opportunities and obstacles define the backdrop for this Delphi study. While risks and barriers are acknowledged throughout, the primary focus lies on solution-oriented requirements and development opportunities as perceived by domain experts.

As part of the project “Linked Care - End-to-end information supply in mobile nursing and care” funded by the Austrian Research Promotion Agency FFG, the University of Applied Sciences FH Campus Wien is researching access to information relevant to nursing and care beyond the boundaries of different care settings. The purpose of the research project is to improve the flow of information and work processes between the various players in the health care system.

### Throughput Model

The throughput model of health services research provides a framework for studying and analyzing health systems [17]. The model helps to place the research question in a broader context by considering the different stages of the health system, from inputs to long-term outcomes. It also makes it possible to understand and analyze the interactions between the different components. In this case, the model helps to structure the research process and assess the relevance of the identified development potentials for health care in the DACHL region. Considering the research question and the heterogeneity of health systems and EHR implementations, this study focused on the dimensions of throughput, output, and outcome.

### Objectives

The purpose of this study was to use the Delphi technique to determine the current status of digital health data exchange in mobile nursing and care in the DACHL region and identify the major challenges and available development potential.

The study aims to answer the following research questions:

1. What requirements and challenges do experts identify for the interdisciplinary and cross-sectoral integration of EHRs in the context of mobile care in German-speaking countries?
2. Which functions and support measures are considered essential by experts for the successful use of EHR systems in mobile care settings?
3. What impact on quality of care, workload, and collaboration can be expected from digital health data exchange in mobile care?

## Methods

### Study Design

This study used a modified 2-round Delphi method. The reporting follows the proposed Delphi reporting guideline by Jünger et al [18]. The Delphi method is a group facilitation technique designed to achieve consensus among a panel of experts through iterative surveys and structured feedback [19]. It also makes it possible to determine and qualify the views of a group of experts on a diffuse issue. The result is the derivation of interventions to respond to the identified problem. However, the identification of divergences can also be seen as a successful result [20]. Methodologically, this Delphi survey is based on a qualitative survey and subsequent quantitative evaluation [19].

### Participants and Recruitment

Experts from research and practice with suitable thematic focuses (mobile nursing and care, digitalization, and health informatics) according to the research question were identified online and invited to participate.

According to Keeney et al [21], it is not the size of the sample that is decisive in Delphi studies but the heterogeneity of the experts. The fact that a high number of experts provides clearer results must be questioned and can even lead to a distortion of the results [22]. In their systematic review of 80 Delphi studies, Boulkedid et al [23] described a median of 17 participants. Based on this, a sample of 15 participants was targeted for this study.

A purposive expert-sampling approach, supplemented by limited snowball sampling to broaden the network, was chosen. All invitees met predefined inclusion criteria and were targeted specifically for their domain knowledge. The inclusion criteria comprised an ongoing professional role in research, project work, industry, or nursing practice with a documented focus on digitalization or digital health, preferably related to mobile care, and legal adulthood (age ≥18 years).

To identify suitable candidates, we combined 4 complementary search streams. First, academic websites of German-speaking universities and universities of applied sciences were screened for departments of nursing, medicine, physiotherapy, occupational therapy, and speech therapy. Whenever a unit listed digital health, health-data exchange, or the digitalization of mobile care as a key focus, the researcher with the highest academic degree in that focus area was shortlisted. Second, an analogous procedure was applied to faculties and institutes specializing in digital health, health informatics, or health IT, and leading vendors of nursing software were contacted to nominate staff with formal digital health qualifications who could provide an industry perspective. Third, project databases (eg, Austrian FFG database) and conference programs (eg, dHealth) were searched for ongoing or recently completed digitalization projects in mobile nursing and care, and project partners with demonstrable digital health expertise were added. Fourth, large providers of home nursing and mobile care were located via a web search. Their public staff pages and LinkedIn profiles helped identify nursing professionals responsible for digital care initiatives, and professional associations were screened for officials overseeing digital health dossiers to ensure frontline and organizational viewpoints.

Two researchers created a master spreadsheet of all candidates. To minimize selection bias, they aimed for a balanced distribution across professional background (medicine, nursing, therapy, IT/industry, theoretical work, and practice expertise), country (Germany, Austria, and Switzerland), and gender, where possible.

Thirty-five experts received a personalized email that included an information letter. Candidates who expressed interest received the informed consent form and were asked to sign and return it before phase 1. People who declined were invited to recommend other qualified colleagues (snowball extension). A total of 19 experts returned a signed consent form and were enrolled. Because this number matched the a priori panel size of 15 participants, no additional recruitment wave was launched.

### Phase 1: Gathering Qualitative Data

Phase 1 involved qualitative interviews conducted using a semistructured interview guide to capture both comprehensive and unanticipated perspectives from participants. The interviews were conducted via Zoom (Zoom Communications) or MS Teams (Microsoft Corp) and were recorded. They lasted between 30 and 60 minutes, and data collection took place between January and April 2024. The interview guide was developed collaboratively by the author team and pretested with a member of the *Linked Care* project team to ensure the clarity, relevance, and feasibility of the questions. The guide is provided in Multimedia Appendix 1. Minor linguistic and structural adjustments were made based on the feedback before data collection began.

Data analysis was conducted using qualitative content analysis according to Mayring [24]. The entire process of coding and category development was carried out in MAXQDA (VERBI GmbH), enabling structured data management and traceability of analytical decisions. The focus of the coding and thematic analysis was deliberately placed on the manifest content level of the data. No interpretive or latent elements were analyzed, as the study aimed to capture and structure the factual expert knowledge and positions expressed during the interviews.

One researcher initially conducted the coding of the interviews. Relevant text segments were assigned to categories developed deductively (based on the interview guide) and inductively (emerging from the interview material). All coded passages were paraphrased to allow abstraction from the original wording and to facilitate systematic comparison. Subsequently, all paraphrased and categorized text segments were reviewed in collaboration with a second researcher to ensure consistency and alignment with the category definitions. This joint review supported the refinement of the category system and increased analytical clarity. Based on the paraphrases, items were then formulated for use in the subsequent quantitative phase. This operationalization process was also conducted by 2 researchers and subsequently discussed to ensure conceptual coherence.

To ensure credibility and confirmability, the entire analysis process, including coding decisions, category assignments, and item development, was systematically documented in an Excel file (Microsoft Corp). An example is presented in Multimedia Appendix 2. All interviews were pseudonymized prior to analysis to minimize bias and ensure a neutral, value-free interpretation. The interviewer and the interviewees had no prior relationship, reducing the potential for influence or role-related bias.

Data analysis began concurrently with data collection, allowing for ongoing reflection and iterative refinement of categories. Reflexivity was addressed throughout the process: both researchers engaged in regular discussions to critically reflect on how their professional backgrounds and assumptions could influence the interpretation of data. The involvement of 2 researchers enhanced both the dependability and confirmability of the findings.

### Phase 2: Two-Round Delphi Online Survey

Following the analysis of the qualitative data, the summarizing categories and subcategories were quantified in a questionnaire.

The questionnaire of the first quantitative survey round comprised 18 topics, each consisting of 3 to 25 items, with 160 items in total. Of these, 159 were statements to be rated on a 4-point Likert scale (“agree,” “tend to agree,” “tend to disagree,” and “disagree”). For 1 item, we provided 14 possible functions of an interdisciplinary EHR named in the interviews and asked participants to rank them in order of perceived importance. An allocation of 12 ranks was possible, whereby functions that were not considered important did not have to be selected. In addition, participants had the opportunity to leave comments in a free-text field after each topic.

In terms of cognitive validation, the concurrent think-aloud method was used [19]. Two participants completed the questionnaire in separate meetings via Zoom. They were asked to share their screens and express their thoughts aloud, and the time required was noted. The testing was accompanied by 2 researchers. The participants gave feedback on the terminology, wording, unclear items, structure of the questionnaire, and visual aspects of the user interface. These participants were not part of the Delphi panel.

After minor revisions to the questionnaire, a link to the online survey (LimeSurvey) was provided to the participants of the first round via email. A reminder was sent 2 weeks later. To reduce bias, the participants’ identities were kept anonymous. The survey was conducted in April and May 2024. A reminder email was sent toward the end of the data collection window. Experts who did not complete the questionnaire despite the reminder were classified as dropouts for this round.

Consensus was defined as ≥75% of the participants rating an item with agreement (“agree” and “tend to agree”) or rejection (“tend to disagree” and “disagree”), which is considered as an acceptable cutoff point [25]. To capture the relative importance of the possible functions, we awarded 12 points for each first rank, 11 points for each second rank, and so on. We calculated the total number of points for each function and the frequency of functions in the top positions. Because the ranking item reflects relative rather than absolute agreement, it was evaluated separately from the consensus analysis, and the ≥75% consensus threshold was therefore not applied to this item.

In the second quantitative round (July and August 2024), only items without consensus were included, as well as the question about possible functions, reduced to the 4 top-prioritized items in the first round. During the analysis of round 1, we identified 1 item, where participants may have misunderstood the question, so we rephrased it and provided this information to the experts. The results of the first round were presented to the participants in percentage and visualized in a bar chart, including all 4 possible answers. The experts were invited to make comments on each item and argue their decision.

As in round 1, a reminder was dispatched shortly before closure, and nonresponders after this reminder were recorded as dropouts. Analysis was performed similar to that in the first round. Due to the alteration of the questionnaire, no measure of response stability was calculated.

The Delphi study was closed after 2 quantitative rounds, as consensus was reached on 88.7% (141/159) of the items, and it is known from the literature that experts often lose interest after 3 surveys [21,26].

### Ethical Considerations

The study adhered to the principles of the Declaration of Helsinki. Under the Guideline for Good Scientific Practice and Ethics in Research of the University of Applied Sciences FH Campus Wien [27], research that involves no medical products or medicinal trials poses no more than minimal risk and gathers only professional opinions from adult participants and is thus exempt from mandatory review by the institutional ethics committee. Because the present Delphi study met all 3 criteria and did not process patient data, formal ethics approval was not required. The project Linked Care, for which the study was carried out, has the Department for Ethics and Law in Medicine, University of Vienna, as an independent consortium partner.

Potential participants received an information sheet and consent form via email. Participation was voluntary. Signed informed consent was obtained before the first interview, and respondents could withdraw at any time without disadvantage. Interview recordings were pseudonymized immediately after transcription, and the reidentification key was accessible only to the first and second authors. Survey response matrices were anonymized. All files were kept on a secure institutional server that was accessible only on password-protected computers. No personally identifying quotations are reported.

Participants received no financial or material compensation for taking part in this study.

## Results

### Overview

**Figure 1:**
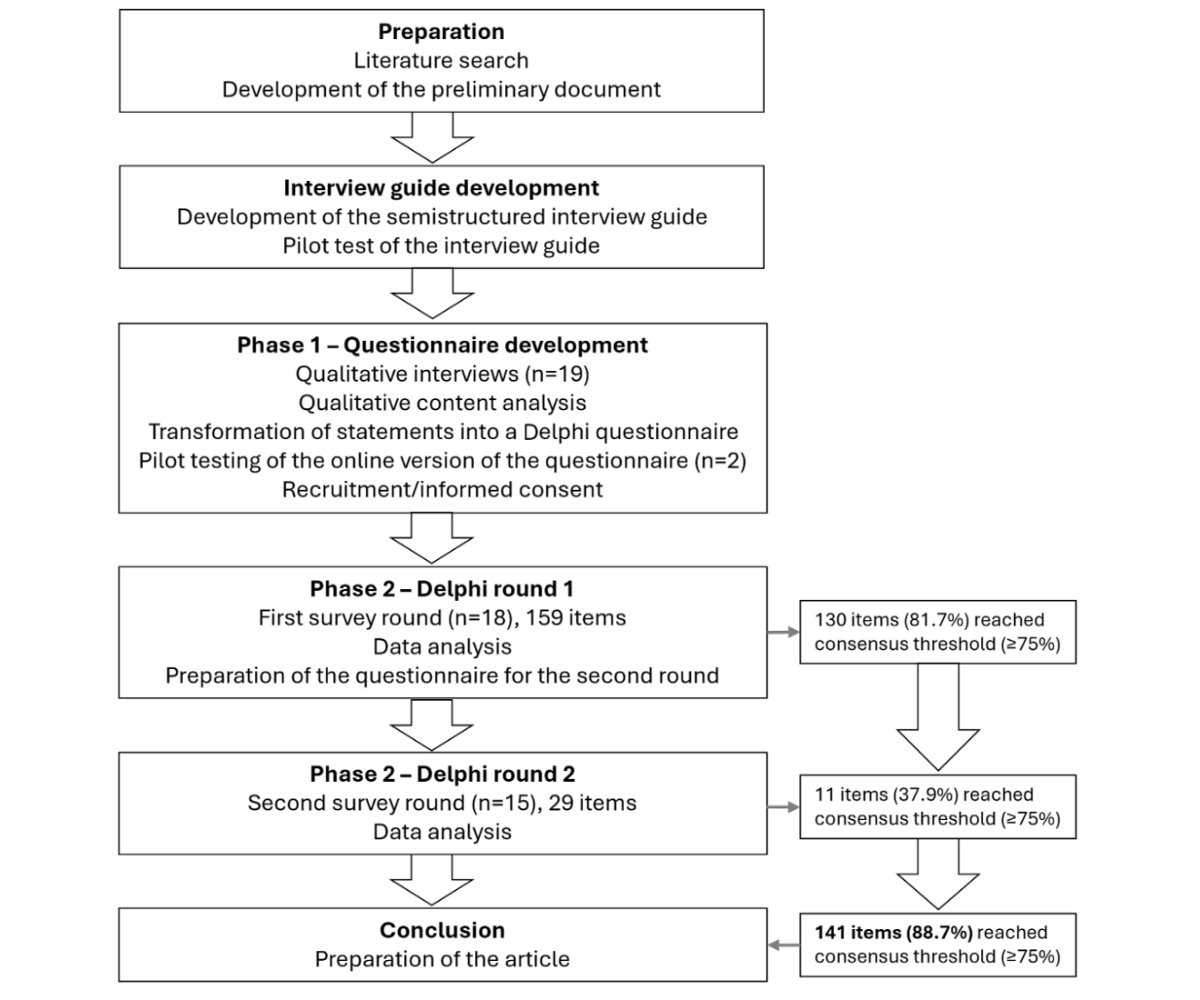
Flowchart of the Delphi process.

We present the characteristics of the participating experts and the results of the Delphi survey. The study flow of the Delphi process is presented in Figure 1.

### Characteristics of the Panel

In total, 19 experts participated in the qualitative interviews, 18 participated in the first quantitative round, and 15 participated in the second round. Demographic characteristics are provided in Table 1. Some dropouts after the first phase were expected, but we were able to retain 78% (15/19) of the interview participants in the second quantitative round.

**Table 1:** Demographic characteristics of the panel.

Variable			Qualitative survey (N=19)^a^		Round 1 (N=18)		Round 2 (N=15)
Age (years), mean (SD)			50.4 (12.3)		48.4 (11.4)		50.9 (13.0)
**Sex, n (%)**							
	Male	11 (58)		10 (56)		9 (60)	
	Female	8 (42)		8 (44)		6 (40)	
**Main country of professional activity, n (%)**							
	Germany	5 (26)		3 (17)		3 (20)	
	Liechtenstein	1 (5)		1 (6)		2 (13)	
	Austria	4 (21)		5 (28)		4 (27)	
	Switzerland	9 (47)		9 (50)		6 (40)	
**Field of expertise^b^, n (%)**							
	Research (nursing research, therapy, and medicine)	7 (37)		10 (56)		9 (60)	
	Technology and IT (digital health, IT, and health informatics)	5 (26)		6 (33)		5 (33)	
	Nursing practice (mobile care setting)	7 (37)		7 (39)		9 (60)	
	Health economics	2 (11)		4 (22)		2 (13)	

^a^Demographic data were not verified during the interviews. The characteristics given here were determined by the researchers based on their research and assumptions; therefore, they may differ from the experts’ self-assessments in the quantitative rounds.

^b^Some participants have several affiliations to different sectors.

### Phase 1

Phase 1 describes the qualitative part of the survey. The results are presented below.

#### Throughput

The expert interviews reveal that a low-threshold connection to the EHR is seen as a crucial starting point for integrating digital health data exchange into mobile nursing. Experts emphasized the importance of software solutions that can seamlessly integrate with existing systems, such as hospital information systems, to simplify the implementation process. Open and interoperable systems were identified as key to enabling data exchange between various stakeholders, such as physicians, nurses, and pharmacies, without requiring new infrastructure.

Agile development processes and iterative approaches were highlighted as essential for tailoring EHR systems to the specific needs of mobile nursing. Flexible structures allow for ongoing optimization, ensuring that digital solutions remain relevant and effective. Standardized terminologies, such as SNOMED CT and LOINC, were considered essential for enabling smooth data exchange and system integration.

Interdisciplinary collaboration was also emphasized. Close cooperation between health care providers and software manufacturers ensures that relevant health data are effectively integrated into patient care, promoting acceptance of new technologies. Furthermore, significant funding for technologies in the health care sector was seen as a prerequisite for the long-term development and maintenance of stable digital systems.

#### Output

The integration of digital tools into mobile nursing has the potential to enhance interdisciplinary collaboration by enabling efficient data exchange among physicians, nurses, therapists, and other professionals. This prevents media discontinuities and optimizes information flow, particularly across different care levels. Moreover, experts highlighted the importance of cross-border data exchange, which is becoming increasingly important for patients in border regions and facilitates access to health care systems abroad.

Staff education and training were frequently mentioned as critical factors in ensuring the effective use of digital systems. Another focus was telemonitoring, given its potential to monitor health parameters in real time and integrate data into EHR systems. This facilitates continuous updates and supports improved care for chronically ill and vulnerable populations in home care settings.

The potential of artificial intelligence (AI) was emphasized, particularly its capacity to enhance efficiency by analyzing large datasets and identifying patterns to support clinical decision-making. However, experts cautioned against risks, such as data misuse, and emphasized the need for clear regulatory frameworks to ensure ethical AI use.

#### Outcome

Digitalization holds significant potential to reduce caregivers’ workload by automating administrative processes and enabling seamless data exchange across care settings. Experts predicted that this may contribute to job satisfaction and address the shortage of skilled workers in mobile nursing. The automation of workflows, particularly in areas, such as medication management, was seen as a way to increase patient safety and reduce error rates.

The seamless integration of health data from outpatient, inpatient, and mobile care settings into EHRs facilitates more comprehensive and coordinated care, particularly for patients with chronic conditions. Additionally, digital documentation enhances transparency, giving patients better access to and control over their personal health information.

Experts also emphasized the potential of international data exchange for improving care, particularly in the context of rare disease research and cross-border health care provision. Digital systems can support crisis management by providing timely and secure access to relevant information, ensuring effective emergency responses and continuity of patient care.

Multimedia Appendix 3 presents a table showing the main results of the analysis in the 3 dimensions of throughput (processes and technologies for integration), output (solutions developed and recommendations), and outcome (long-term impact on caregivers, workflows, and patient care).

### Phase 2

Table 2 presents an overview of the thematic groups, their allocation to the throughput model dimensions, and the proportion of items that reached consensus in the 2 Delphi rounds. Of the 159 items, 141 (88.7%) achieved the defined threshold of 75% agreement or disagreement among participants.

The results of the individual question groups are presented below, structured according to the dimensions of the theoretical model (throughput, output, and outcome).

**Table 2:** Overview of the thematic groups.

Thematic group	Dimension	Items, n	Items with consensus, n (%)
1: General necessity and national requirements	Throughput	10	7 (70)
2: Economic aspects	Throughput	4	4 (100)
3: Financing	Throughput	3	3 (100)
4: Research and development	Throughput	14	14 (100)
5: Effects of interdisciplinary data exchange	Outcome	25	25 (100)
6: Implementation and evaluation	Throughput/outcome	12	9 (75)
7: Data authorization	Throughput	22	15 (68)
8: Relevant functions of an EHR^a,b^	Output	—^c^	—
9: Telemonitoring	Output	4	4 (100)
10: Usability and support	Output	3	3 (100)
11: Obligations and freedom of choice	Throughput	7	5 (71)
12: Digitalization in everyday health care	Throughput	9	8 (89)
13: Standardization, classification systems, and terminologies	Throughput/outcome	12	11 (92)
14: Role of AI^d^	Output/outcome	2	2 (100)
15: Training and interdisciplinary teamwork	Output	4	4 (100)
16: Data protection	Throughput	7	7 (100)
17: Data exchange in disaster and crisis situations	Outcome	4	4 (100)
18: International health data exchange	Output	17	16 (94)
Overall	—	159	141 (89)

^a^EHR: electronic health record.

^b^Item was assessed via ranking and therefore not included in the item count or consensus calculation.

^c^Not applicable.

^d^AI: artificial intelligence.

#### Throughput

##### General Necessity and National Requirements

The study emphasized that the digital exchange of health data is of great importance for mobile care and support. Most of the participants (17/18, 94%) consider networked health data to be essential for ensuring the continuity of care. The integration of such data into EHRs was also considered a key factor by 83% (15/18) of participants.

However, there were mixed views on the political framework conditions. While in the first quantitative round of the survey, only 35% (6/17) of participants were of the opinion that existing framework conditions were sufficient for the implementation of interdisciplinary and intersectoral EHRs, this number rose to 67% (10/15) in the second round. The results differ depending on the country. In Austria, all participating experts (4/4, 100%) stated in the second round that the political conditions were in place. Experts from Germany and Switzerland, on the other hand, still saw room for improvement in their countries.

There was broad agreement on the assessment of legal requirements. These are described by a large majority (13/15, 87%) as being frequently impractical. Additionally, 94% (16/17) argued that the state should primarily set the regulatory framework conditions instead of developing information systems itself. There was also consensus regarding the interoperability of software solutions from different providers, which all respondents (18/18, 100%) described as absolutely necessary.

##### Economic Aspects and Financing

The majority of experts (14/17, 82%) were in favor of involving the private market to a greater extent to drive innovation in the health care sector. At the same time, it was emphasized that clear approval procedures are needed to ensure reliable standards for EHRs. However, the possibility of choosing between different EHR providers was viewed skeptically. In the second round of the survey, approval on this point fell noticeably from 44% (8/18) to just 21% (3/14). The decline may reflect concerns about potential system fragmentation.

Another key point of discussion was the role of the state in financing. A clear majority of 94% (16/17) supported the view that the state should assume financial responsibility, particularly with regard to regulating data flows and setting binding standards. At the same time, 77% (13/17) of respondents stated that they consider the financial interests of software providers to be a potential obstacle to standardization.

##### Research and Development

The involvement of various stakeholders was rated as necessary by 100% (18/18) of experts, including doctors, nurses, and patients. Meeting the demand for needs-oriented technological developments was viewed more critically, with 82% (14/17) of experts not considering current developments to be sufficiently aligned with actual care needs.

##### Implementation and Evaluation

The experts were largely in agreement that the additional costs incurred by the introduction and operation of EHRs must be compensated for all health care professionals involved. This demand received 88% (14/16) agreement. Similarly, 93% (14/15) of experts emphasized that access to EHRs must be made as simple and unbureaucratic as possible for health care providers.

The discussion was more controversial when it came to the question of whether financial incentives are necessary to encourage health care professionals to participate in EHRs. While 63% (10/16) agreed in the first round, this figure fell to 33% (5/15) in the second round.

A particularly important result was the requirement that citizens must clearly understand the benefits of an interdisciplinary and intersectoral EHR to create acceptance, with 94% (16/17) of experts supporting this point. In addition, all agreed (17/17, 100% agreement) that the trust of the population in such systems is of central importance.

The development in the comparison of trust in digital documentation versus paper documentation is also worth mentioning. While there were still doubts in the first round, the rejection of the statement that paper documents enjoy more trust increased in the second round, and 87% (13/15) of experts stated that digital systems are perceived as more trustworthy.

##### Data Authorization

The question of access rights to health data within an interdisciplinary EHR led to one of the most controversial discussions among the experts. This was the area with the lowest level of consensus among the experts. This illustrates the great need for clear regulations and a differentiated discussion of this topic.

Emergency doctors received the highest level of approval (16/16, 100%) for access rights, while general practitioners (GPs), specialists, and registered nurses also achieved high approval ratings of 94% each, which underlines their central position in patient care. Ambulance workers (14/16, 88%), physiotherapists, and psychologists (13/16, 81%) were also considered by the majority of experts to be authorized to access health data.

There was less agreement on access rights for nursing assistants and health care assistants, with 63% (10/16) agreeing in the first round and 60% (9/15) agreeing in the second round. Access for home help and 24-hour caregivers was even more controversial. Here, approval for both professions was at a low of 31% (5/16) in the first round and dropped further to 20% (3/15; home help) and 23% (3/13; 24-hour caregivers) in the second round.

The experts were also restrained when it came to administrative staff in health care facilities. Approval for this group fell from 44% (7/16) in the first round to 33% (5/15) in the second round.

In contrast, there was greater agreement regarding the institutions. There was 93% (14/15) approval regarding health insurance companies being given the right to access anonymized or pseudonymized data. For private insurance companies, however, the experts tended to be more reserved, with 60% (9/15) being against access. The experts rated access to anonymized data by research institutions as much more important, with 94% (15/16) of respondents being in favor.

##### Obligations and Freedom of Choice

In this area, the majority of experts agreed that it should be mandatory for all health care providers to be linked to a state-regulated EHR (15/16, 94% agreement). This was considered necessary to ensure a smooth exchange of information. For patients, the opt-out solution, automatic participation with the option of actively opting out, received broad support (11/12, 92%) from the experts, as it is considered more feasible for a high participation rate than the opt-in alternative, which requires active registration for the EHR.

However, the question of whether patients who refuse the EHR should pay for the additional information management costs remains controversial. Although agreement rose slightly to 60% (9/15), the result remained ambiguous. The experts were particularly critical of the question of state access to stored health data. While 41% (7/17) agreed in round 1, this figure fell to 33% (5/15) in round 2.

The assessment of support for vulnerable groups was much clearer, with 100% (15/15) of experts calling for simple and secure options for transferring the management of the EHR to a trusted person if patients are unable to do so themselves.

##### Digitalization in Everyday Health Care

Experts unanimously agreed (15/15, 100%) that successful digital transformation requires rethinking health care processes rather than simply digitizing existing analog workflows. Likewise, 100% (14/14) of participants emphasized the importance of agile development structures to enable flexible and rapid adjustments in the implementation of interdisciplinary and cross-sectoral EHRs.

All experts (15/15, 100%) favored a nationwide rollout of such EHRs over regional pilot projects. The strict separation between inpatient and outpatient care was seen as a barrier to digital integration by 93% (14/15) of experts. In addition, 93% (14/15) agreed that interdisciplinary and intersectoral documentation can help counteract fragmentation in the health care system.

Support for integrating existing digital data flows into future solutions increased between rounds, from 69% (9/13) in the first round to 87% (13/15) in the second round. This highlights a growing recognition of the importance of continuity and technical compatibility in digital system development.

##### Standardization, Classification Systems, and Terminologies

The experts emphasized the central role of standardization and reference terminologies for the interoperability of interdisciplinary EHRs. There was a broad consensus (13/14, 93%) to rely on existing classification systems to enable consistent and comprehensible data exchange, with 75% (9/12) of experts considering the use of Snomed CT to be adequate in the care context. The need for mapping tables to translate different systems was emphasized (10/13, 77% agreement), although there was no consensus on the extent to which this could result in information loss. In addition, 83% (10/12) of experts considered the combination of structured data and free text to be necessary in order to ensure the individuality of the care process and to map all relevant information.

##### Data Protection

The experts emphasized that data protection must be designed in such a way that it does not impede research (14/14, 100% agreement) while upholding the highest security standards against cybercrime (13/15, 87%). According to the experts, the relevance and validity of the stored data must be ensured, while too much or irrelevant information should be avoided in order to maintain clarity (11/12, 92% agreement). Clearly defined rules on accessing, storing, and deleting data are essential, according to the participants (15/15, 100% agreement). In addition, 77% (10/13) of experts saw a need to precisely define the obligations associated with the availability of large amounts of data.

#### Output

##### Relevant Functions of an EHR

The experts prioritized 14 potential EHR functions in a 2-step ranking exercise. In round 1, each participant could distribute the ranks 1-12 to those functions they considered important (unselected items received no points). Diagnoses (162 points) and the medication sheet (150 points) led the field, followed by anamnesis (134 points) and assessments including risk factors (125 points). In fifth place was the transfer of prescriptions from GPs to pharmacies (108 points). Patient monitoring was in sixth place (106 points), while workflow-oriented tools, such as an interdisciplinary calendar (66 points; rank 8) and a messenger (27 points; rank 13), scored considerably lower.

In round 2, the panel was asked to rerank only the 4 highest-scoring functions from round 1 in order to confirm or reorder this “short list.” The top positions remained stable: diagnoses again ranked first (32/48 possible points), followed by medication sheet (27 points), assessments (19 points), and anamnesis (18 points).

##### Usability and Support

The results indicate that ease of use and good support are crucial for the successful introduction of interdisciplinary EHRs. The experts agreed that user friendliness is an indispensable prerequisite (17/17, 100% agreement) and should be tested rigorously to meet its practical requirements. Moreover, 82% (14/17) emphasized that different user interfaces for health care providers and citizens are necessary to address the different needs of user groups. Furthermore, it became clear that reliable, comprehensive support is essential for both professionals and citizens. Without this support, according to the experts, broad acceptance and smooth use of the systems are hardly conceivable.

##### International Health Data Exchange

The results showed that international health data exchange is seen as increasingly important by experts, particularly in light of increasing patient mobility in Europe. Among the participants, 93% (13/14) saw clear benefits of cross-border data exchange in migration and tourism, especially for patient safety. Unified European standards for data exchange, data processing, and data protection were rated with high approval (12/13, 92%), while global standards received less support (4/14, 29% approval).

The majority of experts (13/15, 87%) were in favor of pushing ahead with national developments and taking EU standards into account in parallel. In the context of mobile care, 100% (14/14) of experts agreed on the need for international networking.

Cross-border access to important health information, such as vaccination status (11/13, 85% agreement) and infectious diseases (10/13, 77% agreement), was also seen as relevant in order to be able to react quickly in crisis situations. The experts also emphasized the need for clear regulations on the anonymization of data for international research (14/14, 100% agreement) and recognized the potential for generating knowledge from extensive data sets.

##### Training and Interdisciplinary Collaboration

The experts agreed that existing hierarchies in the health care system must be broken down in order to enable smooth cooperation between the various professional groups (13/13, 100% agreement). To this end, it is important that more knowledge about the skills of all those involved in health care is provided in training programs to enable communication between professional groups on an equal footing (15/15, 100% agreement). The need to integrate digital skills more strongly into the curricula of health care professions was also emphasized in order to prepare professionals for the challenges of digitalization. Furthermore, the majority of experts (13/14, 93%) were in favor of placing a stronger focus on applied research in order to develop practical solutions that can be implemented directly in day-to-day care.

##### Telemonitoring and the Role of AI

The experts emphasized the importance of telemonitoring for mobile patient care and digital data exchange. Effective telemonitoring requires the exchange of medical and nursing data as well as clearly defined standards for health care applications (17/17, 100% agreement). Measured values, such as blood pressure and blood sugar, should be integrated directly into EHRs in order to improve the care of chronically ill patients and make processes in mobile care more efficient.

Regarding AI, 91% (10/11) of experts agreed that it could be particularly useful for structuring free-text information in order to facilitate data exchange. However, it was clearly emphasized that AI may only provide suggestions for decisions, while the final decision must always be made by a human expert.

#### Outcome

Some topics assigned to the outcome dimension, such as standardization of data formats, the use of AI, and aspects of implementation and evaluation, have already been addressed in the throughput and output sections. This is because these areas are not only linked to the long-term effects of digital health data exchange but also closely tied to the development, implementation, and functional design of interdisciplinary EHRs. For example, standardization plays a critical role in enabling interoperability (throughput), contributes to user acceptance and integration into care processes (output), and ultimately influences the quality and continuity of care (outcome).

Therefore, while these topics are analytically situated within the outcome dimension, highlighting their potential long-term impact on care quality, patient safety, and professional collaboration, they were necessarily discussed earlier in the context of structural and functional prerequisites. The following subsections focus on outcome-specific results that reflect the anticipated effects of interdisciplinary digital documentation, particularly with regard to interprofessional cooperation and data availability in disaster and crisis situations.

#### Effects of Interdisciplinary Data Exchange

The question group addressing the effects of interdisciplinary digital documentation had a high level of agreement overall, with 100% (17/17) of participants in both rounds agreeing that such documentation enables professionals to gain a holistic view of patients with minimal effort and improves intraprofessional handovers. The potential to reduce transcription and transfer errors (17/17, 100%) and decrease duplication of examinations (17/17, 100%) was also fully endorsed.

Strong agreement was recorded for the contribution of digital documentation to improving medication safety (16/16, 100%), enhancing interprofessional collaboration (16/16, 100%), and enabling goal-oriented interdisciplinary care (17/17, 100%). The majority also agreed that it increases patient safety (15/17, 88%), reduces redundant data collection (16/17, 94%), and improves care quality in mobile settings (15/16, 94%). Regarding its potential to reduce administrative workload and relieve nursing staff, 82% (14/17) of experts agreed. Similar levels of support were seen for its contribution to increased time for direct patient interaction (13/17, 77%) and addressing the shortage of skilled personnel (13/17, 77%).

In terms of system-level effects, 87% (13/15) of participants in the second round expected financial relief for the health care system (up from 67% [10/15] in the first round). The potential to foster mutual oversight between professions was also acknowledged, with 80% (12/15) agreement in round 2.

#### Data Exchange in Disaster and Crisis Situations

The experts emphasized the importance of fast and reliable access to health data in crisis situations. A large majority (10/11, 91%) consider national data exchange to be essential in order to remain capable of acting in emergencies, while 75% (9/12) also emphasized international exchange in cross-border crises.

To ensure the continuity of care during technical disruptions, 93% (14/15) of participants supported the availability of alternative solutions to bridge outages or blackouts. Furthermore, all participants (15/15, 100%) agreed on the necessity of clear regulations for data access during emergencies, enabling health care providers to access critical information even under challenging conditions.

**Figure 2:**
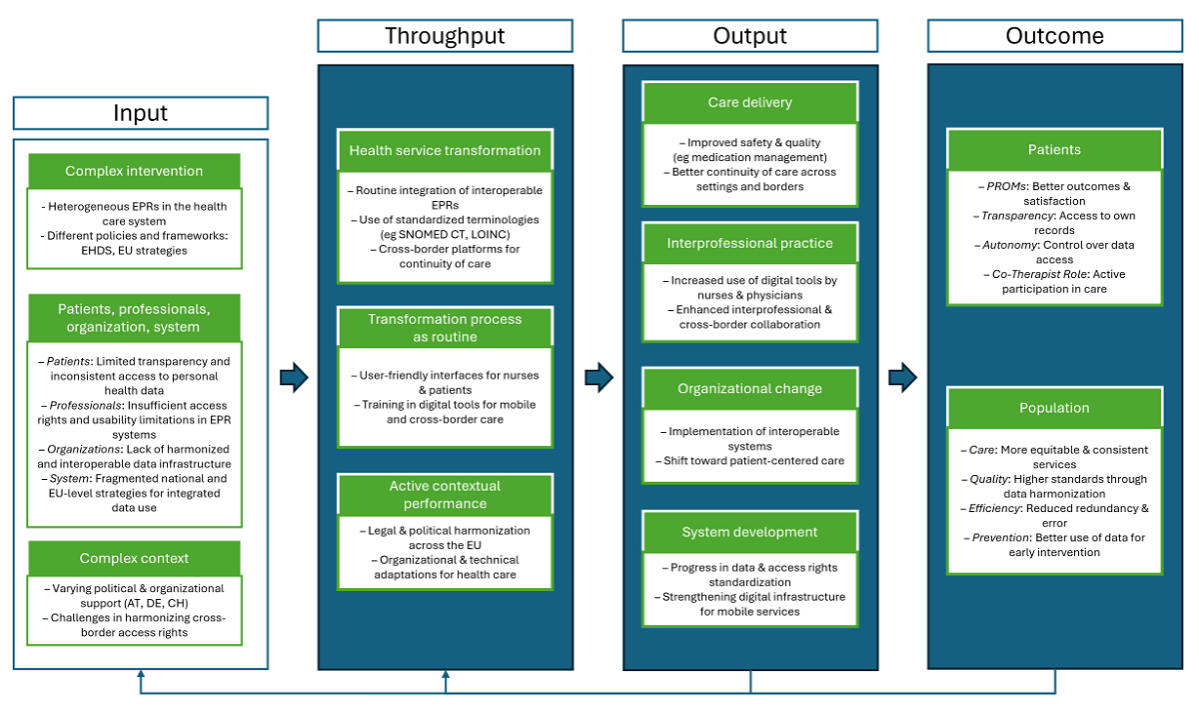
Visual summary of the Delphi study results, structured according to the input-throughput-output-outcome model by Pfaff et al [17]. Input refers to the current digital infrastructure, roles, and contextual conditions. Throughput captures technical, legal, and organizational requirements for interdisciplinary data exchange. Output reflects short-term effects such as usability and collaboration. Outcome describes long-term impacts on care quality, patient participation, and system efficiency. AT: Austria; CH: Switzerland; DE: Germany; EHDS: European Health Data Space; EPR: electronic patient record; LOINC: Logical Observation Identifiers, Names, and Codes; PROMs: patient-reported outcome measures; SNOMED CT: Systematized Nomenclature of Medicine–Clinical Terms.

Figure 2 provides a consolidated visual representation of the study results based on the input-throughput-output-outcome model [17]. It summarizes the key thematic findings derived from both qualitative and quantitative rounds and illustrates their classification across the 4 dimensions.

## Discussion

### Overview

This Delphi study aimed to identify the requirements and challenges for the interdisciplinary and cross-sectoral integration of EHRs in mobile care within the German-speaking DACHL region. It also examined which functions and support structures are considered essential for the effective use of EHR systems in mobile care settings. In addition, it investigated the impact that digital health data exchange can have on quality of care, workload, and professional collaboration, and situated these results within the broader scientific and policy discourse.

The findings provide a differentiated picture of the current situation, highlighting technical, regulatory, and practical barriers, as well as pointing toward concrete development opportunities. Key topics included the need for standardized, interoperable systems, clear and differentiated access rights, and a strong focus on usability and support. These issues are particularly relevant in mobile care contexts, where fragmented data structures and unclear access policies can significantly impair the continuity of care.

Throughout the discussion, the findings are interpreted through the lens of the input-throughput-output-outcome model by Pfaff et al [17], which serves as a helpful framework for understanding how structural, procedural, and outcome-oriented aspects of digital health interact in complex care environments.

### Access Rights and the Role of Nursing Assistants

The Delphi study revealed disagreement regarding access rights for nursing assistants, with only 31% (5/16) of experts supporting their inclusion, in contrast to the strong consensus for granting access to registered nurses and physicians (15/16, 94%). This disparity highlights a critical gap in the integration of nursing assistants into interdisciplinary care frameworks, particularly in mobile and home care settings.

However, several studies emphasize that nursing assistants, as frontline providers, often play a central role in patient care by observing early signs of deterioration and reporting clinical changes. Sund-Levander and Tingström [28] demonstrated that nursing assistants contribute essential insights to clinical assessments, especially in older populations, yet their observations often lack formal documentation channels.

To resolve this, recent literature calls for context-sensitive and role-based access policies. Sarkar [29] emphasized that access should be linked to the specific task and care situation: the right person with the right data at the right time. In the context of public health emergencies, Lee et al [30] similarly advocated for sociotechnical infrastructure that can support timely, purpose-bound access to data across institutional boundaries.

While national regulations in the DACHL region provide the overarching legal frameworks for data access, they do not define uniform role-based entitlements for individual professional groups. Especially for assistant-level roles, such as nursing assistants, current legislation remains vague or restrictive. Within this legal scope, health care organizations often bear the responsibility for implementing internal access rights policies. Role-based access control models can support these efforts by aligning data access with actual job responsibilities while ensuring that technical and legal safeguards are upheld [31,32].

The discrepancy between the Delphi results and the perspective in the literature underscores the need for further research and policy development in this area. While the Delphi panel expressed hesitancy toward broader access for assistant roles, existing evidence highlights their relevance in care continuity and team-based workflows. Busse et al [33] emphasized that the design of digital systems and access policies must involve all relevant professional groups to ensure that access is ethically sound, practically applicable, and aligned with real-world care responsibilities.

To bridge this gap between regulatory limitations, organizational discretion, and the practical needs of care delivery, future strategies should incorporate structured stakeholder engagement and empirical evaluation. Only through inclusive and evidence-informed processes can access rights be defined in a way that is both secure and supportive of high-quality, interdisciplinary care.

### Cross-Border Data Exchange and Mobile Care

Our Delphi panel largely concurred with the literature and current policy initiatives on the value of cross-border data sharing. Nearly all experts (13/14, 93%) viewed seamless exchange of health information as essential for patient safety, crisis response, and care accessibility, especially in border regions, migration settings, and tourism. This mirrors the findings of Li et al [1], who identified interoperability as pivotal to care coordination and safety, and Saranto et al [14] and Taneva et al [13], who stressed that standardized nursing documentation underpins the continuity of care. Consistently, up to 92% (12/13) of our panel supported EU-wide documentation standards.

These views align with the EHDS, which advocates secure, standardized exchange across member states to advance both clinical practice and research. All panelists (14/14, 100%) endorsed clear rules for data anonymization to facilitate international research and knowledge generation. Nonetheless, structural barriers persist. Divergent national laws, heterogeneous technical infrastructure, and nonaligned nursing terminologies hinder truly seamless exchange. Overcoming these gaps will require harmonized governance, targeted investment in interoperable systems, and a clear delineation of professional responsibilities at the transnational level.

### System Design, Usability, and Standardization

Interoperability remains one of the most pressing and unresolved challenges in digital health and nursing, not only from a technical standpoint but also regarding clinical integration, workflow alignment, and administrative governance. Recent literature highlights that data in EHRs are often poorly structured and insufficiently standardized, limiting semantic interoperability across systems. Although international coding standards, such as SNOMED CT, LOINC, and CCC, are available, their adoption is inconsistent, and many systems lack adequate interfaces to enable meaningful cross-institutional data exchange. Furthermore, technical barriers are compounded by clinical challenges: nursing-specific content is often not fully integrated into EHRs, hindering comprehensive interprofessional collaboration and leading to fragmented care delivery. Moreover, workflows in nursing are rarely aligned with the design of digital systems. Usability issues, limited training, and time constraints encourage continued use of unstructured documentation formats, thereby undermining the interoperability of data at the point of care. On an institutional level, decentralized governance structures, diverging stakeholder interests, and inconsistent legal frameworks further obstruct harmonized data management and sharing [4].

Against this backdrop**,** our Delphi panel highlighted design features that can mitigate these barriers. All participants agreed that user-friendliness must be a core design criterion. This aligns with existing literature emphasizing usability as a critical factor in EHR adoption and use, especially in nursing [13,14]. Experts further highlighted the importance of standardized terminologies (eg, SNOMED CT) and process-oriented implementation. Systems should not merely digitize paper workflows but support new, efficient care processes. Agile development, modular adaptability, and tailored user interfaces for different user groups (eg, professionals vs patients) were also emphasized.

The experts unanimously emphasized the need for binding, standardized data formats. Yet, recent implementation research paints a more sobering picture. In a recent mixed-methods study examining Germany’s digital care transition record and its transmission processes [34], the authors argued that as long as the adoption of digital standards is voluntary and lacks binding regulatory support, the establishment of a uniform data format will be considerably delayed, and without stronger enforcement and broad stakeholder commitment, the goal of seamless care transition is unlikely to be achieved [34].

The Delphi panel identified diagnoses, medication documentation, assessments, and medical history as the most essential EHR functions. This prioritization corresponds with findings from an international comparative analysis of 9 EHR systems across 7 countries, which identified core functionalities, such as medication documentation and medical history, as consistently implemented features [35].

By contrast, telemonitoring or patient monitoring illustrates how perceived usefulness and implementation priority can diverge. Although all experts unanimously agreed in the Likert block that telemonitoring data should be integrated into EHRs (17/17, 100% agreement), the function reached only rank 6 in the priority list.

These findings reinforce the need for collaborative system development involving frontline professionals and IT experts. To avoid misaligned system features or underutilization, digital infrastructure must evolve in tandem with the practice environments they are meant to support.

### Limitations

This study has some limitations. First, expert recruitment was based on purposive sampling and conducted by the researchers through online searches and professional networks. While this method enabled the inclusion of individuals with demonstrated expertise in mobile care, digital health, and health informatics, it inherently reflects subjective researcher judgment. Moreover, all invitees operate within the German-speaking DACHL region, and thus, their views are embedded in a common regulatory and reimbursement environment. Applicability to settings with different legal frameworks or care models therefore remains uncertain. The digital-health focus of our search further favored the recruitment of technology-affine experts, while the perspectives of data-protection advocates, payers, or patient representatives were not captured, introducing a potential optimism bias toward technical feasibility. Finally, the purposive approach yielded a relatively small panel including drop-outs. Because the survey was conducted under full anonymity, individual responses could not be linked across Delphi rounds. Consequently, nonresponse bias was assessed at the panel level by comparing the distribution of key demographic and professional attributes (age, sex, main country of activity, and field of expertise) across the initial sample (n=19), round 1 (n=18), and round 2 (n=15) (see Table 1). No conspicuous shifts toward any single subgroup were observed. In addition, overrecruitment ensured that the predefined target panel size (n=15) was retained despite attrition. Nonetheless, because individual response patterns could not be tracked, a residual attrition bias cannot be completely ruled out. Therefore, the findings are not generalizable but rather reflect a consensus among a selected group of stakeholders with domain-specific knowledge.

Second, due to the close professional and geographic proximity within the German-speaking DACHL region, several experts reported difficulty in assigning themselves to a single country or disciplinary field. This overlap reflects the interwoven nature of academic and practical work in the region but may have blurred distinctions between national perspectives or disciplinary boundaries.

Third, the first-round questionnaire was extensive and may have led to reduced engagement in later rounds. Although 78% of the initial participants completed the second round, the depth and consistency of responses may have been affected by survey fatigue.

Lastly, while a third Delphi round could have allowed for further refinement of certain items and feedback loops based on round 2 comments, the study was concluded after 2 rounds. This decision was supported by the high overall level of consensus (141/159, 88.7%) and by prior evidence indicating reduced expert engagement beyond the second round.

### Implications and Future Directions

Building on the key findings of this Delphi study, several implications for policy, system development, and future research can be identified. A central takeaway is the need to shift from static digital documentation toward interoperable, agile systems that are tailored to the dynamic workflows of mobile care. Rather than replicating analog processes, EHR systems must actively support coordination, flexibility, and real-time information access. This requires regulatory frameworks that clearly define access rights while maintaining high standards of data protection.

The prioritization of core functions, such as medication documentation, diagnostic summaries, and structured assessments, reflects the importance of focusing on clinically relevant features that enhance the continuity of care. To ensure practical usability, digital tools must be developed with direct input from nurses, care professionals, and other frontline staff.

Another key implication is the necessity of integrating all care-relevant roles, including nursing assistants, into access protocols. Their operational role in mobile and home care settings makes them critical for ensuring uninterrupted care delivery.

Finally, future studies should move beyond expert consensus and examine how interdisciplinary EHRs affect real-world care delivery. Longitudinal research and pilot implementations are needed to assess system performance, user satisfaction, and the potential for unintended consequences, particularly in cross-border or decentralized care contexts.

### Conclusion

This Delphi study provides a comprehensive expert-based assessment of the requirements, challenges, and expected effects associated with the interdisciplinary and cross-sectoral implementation of EHRs in mobile care settings in German-speaking countries. The findings highlight the need for interoperable, standardized, and user-centered digital systems that reflect the realities of mobile care.

Experts emphasized that the successful adoption of EHRs depends on process-oriented implementation strategies, clear access rights, and strong support structures for both users and system maintenance. Prioritized functions, such as medication documentation and diagnostic data, were seen as essential for improving care continuity and patient safety.

In addition, the study underscores the importance of integrating mobile care providers, including nursing assistants, into digital care infrastructure to avoid fragmentation and optimize interprofessional collaboration.

By identifying consensus-based focus areas and barriers, this study contributes to ongoing national and European discussions on digital health transformation and offers actionable insights for system design, regulation, and further research. Addressing the highlighted issues will be critical for advancing safe, efficient, and equitable digital health data exchange across all care settings.

## Appendix

Multimedia Appendix 1 Guide for the qualitative interviews.

Multimedia Appendix 2 Example for the development of the quantitative questionnaire based on the qualitative interviews.

Multimedia Appendix 3 Dimensions and key findings from the qualitative survey (phase 1) and thematic groups.

## Abbreviations

AI: artificial intelligence
CCC: Clinical Care Classification
EHDS: European Health Data Space
EHR: electronic health record
ELGA: Elektronische Gesundheitsakte
ePA: elektronische Patientenakte
EPD: elektronisches Patientendossier
EU: European Union
GP: general practitioner
LOINC: Logical Observation Identifiers, Names, and Codes
SNOMED CT: Systematized Nomenclature of Medicine–Clinical Terms

## Data Availability

The anonymized response matrices and aggregated demographic summaries generated and analyzed in this study are available from the corresponding author for noncommercial research purposes on reasonable request. Individual-level demographic records (age, sex, country, and field of expertise) are not shared, as the small, highly specialized panel could be reidentified through unique attribute combinations. Access to the available data requires the signing of a data use agreement that (1) limits use to the stated scientific purpose, (2) prohibits any attempt at reidentification, and (3) ensures compliance with the European Union General Data Protection Regulation and institutional data protection guidelines. Full interview transcripts are not publicly shared because complete anonymization cannot be guaranteed.
