# Supplementary material for: The Requirements and Development Potential of Interdisciplinary Digital Health Data Exchange in Mobile Nursing and Care Settings in German-Speaking Countries: Delphi Study
Source: J Med Internet Res. 2025 Aug 13;27:e78193. doi: 10.2196/78193 (PMC12391839; doi:10.2196/78193)
Supplement: Multimedia Appendix 1 [file jmir_v27i1e78193_app1.docx]

Multimedia Appendix 1. Guide for the qualitative interviews.

| **Deutsch (Original)** | **Englisch (Übersetzung)** |
| --- | --- |
| **Standortbestimmung**  Ich möchte mit Ihnen über den digitalen Gesundheitsdatenaustausch im mobilen Pflege- und Betreuungssetting sprechen. Könnten Sie mir kurz den aktuellen Stand der digitalen Unterstützungsangeboten in der mobilen Pflege und Betreuung in ihrem Land schildern?  Welche Technologien, Infrastrukturen und rechtlichen Rahmenbedingungen gibt es in Ihrem Land?  Wie werden diese umgesetzt?  **Entwicklungspotential, Herausforderungen**  Wo sehen Sie die Notwendigkeit für einen verstärkten digitalen Gesundheitsdatenaustausch in Ihrem Land/Bundesland?  Wie könnte dies umgesetzt werden? Strategien, Innovationen, Technologien?  Mit welchen Herausforderungen im Rahmen der Digitalisierung ist das mobile Pflege- und Betreuungssetting Ihrer Meinung nach konfrontiert? | **Status Assessment**  I would like to speak with you about the digital exchange of health data in home-based and mobile care settings. Could you briefly describe the current state of digital support services in mobile care and support in your country?  Which technologies, infrastructures, and legal frameworks exist in your country?  How are these being implemented?  **Development Potential and Challenges**  Where do you see the need for an enhanced digital exchange of health data in your country/federal state?  How could this be implemented? Strategies, innovations, technologies?  In your opinion, what challenges does the mobile care and support sector face in the context of digitalisation? |
| **Priorisierung Ö – Vergleich mit TN Länder**  In einer nationalen Erhebung in Österreich wurden folgende drei Bedarfe zur *Verbesserung des Informationsflusses im mobilen Pflege- und Betreuungssetting* identifiziert: Digitale Gesundheitsplattform inkl. Middleware, interdisziplinäre und intersektorale Dokumentation, eMedikation/eVerordnung. (Anm.: werden erklärt) Bitte erläutern Sie, ob und inwiefern diese Themen auch *in Ihrem Land/Bundesland* eine Rolle spielen.  Gibt es in ihrem Land bereits dazu Forschungsprojekt?  Haben Sie Empfehlungen zur Umsetzung dieser Bedarfe? | **Prioritisation in Austria – Comparison with Other Countries** A national survey in Austria identified the following three needs to improve information flow in mobile care and support settings:  A digital health platform including middleware; Interdisciplinary and intersectoral documentation; Electronic medication management/ePrescriptions (Note: These will be explained.)  Please explain whether and to what extent these topics also play a role in your country/federal state. Are there already research projects related to these topics in your country? Do you have any recommendations for the implementation of these needs? |
| **Grenzübergreifender Verfügbarkeit**  Auf europäischer Ebene wird die Vernetzung zum Austausch von Gesundheitsdaten angestrebt.  Sehen Sie einen Bedarf für die grenzübergreifende Verfügbarkeit von Gesundheitsdaten?  Bei „Ja“: Welche gesundheitsbezogenen Informationen sollten international verfügbar sein, aus ihrer Sicht?  In welchen Bereichen (der Versorgung) ist der grenzüberschreitende Gesundheitsdatenaustausch für die mobile Pflege und Betreuung relevant?  Welches Potential hätte der internationale digitale Gesundheitsdatenaustausch in der mobilen Pflege und Betreuung für die Versorgungsleistung?  Welches Outcome/Wirkung erwarten Sie sich davon? | **Cross-Border Availability** At the European level, there is an effort to enable cross-border exchange of health data. Do you see a need for the cross-border availability of health data? If yes: In your opinion, which health-related information should be available internationally?  In which areas of care is cross-border health data exchange relevant for mobile care and support? What potential does international digital health data exchange hold for the quality of care in mobile settings? What outcomes or impacts would you expect from this? |
