# Supplementary material for: The Requirements and Development Potential of Interdisciplinary Digital Health Data Exchange in Mobile Nursing and Care Settings in German-Speaking Countries: Delphi Study
Source: J Med Internet Res. 2025 Aug 13;27:e78193. doi: 10.2196/78193 (PMC12391839; doi:10.2196/78193)
Supplement: Multimedia Appendix 2 [file jmir_v27i1e78193_app2.docx]

Multimedia Appendix 2. Example for the development of the quantitative questionnaire based on the qualitative interviews.

|  | **Expert Interview** | **Paraphrase** | **quantitative Survey** |
| --- | --- | --- | --- |
| German (original) | *Klar, durch den Tourismus, durch so große Fluchtbewegungen, Stichwort Ukraine oder auch hier so die die auf jeden Fall. Da gibt es schon sehr sehr großen. (..) Ähm, ja, grenzüberschreitenden Verkehr. Und es sind viele ausländische Patienten auch, die hier behandelt werden und andersrum ja auch, dass wir im Ausland behandelt werden als Deutsche.* | In Zukunft werden mehr Personen aus dem Ausland im Inland betreut, dadurch wird der grenzüberschreitende Datenaustausch immer wichtiger. | In Zukunft ist mit verstärkter Patient*innenmobilität zu rechnen, weshalb der internationale Gesundheitsdatenaustausch immer wichtiger wird. |
| English (free translation) | [Of course, through tourism, through such large refugee movements, keyword Ukraine or also here like that in any case. There's already a very, very big one. (...) Um, yes, cross-border traffic. And there are also many foreign patients who are treated here and vice versa, that we are treated abroad as Germans.] | [In the future, more people from abroad will be cared for in our country, making cross-border data exchange increasingly important.] | [Increased patient mobility is to be expected in the future, which is why international health data exchange will become increasingly important.] |
