# Supplementary material for: The Requirements and Development Potential of Interdisciplinary Digital Health Data Exchange in Mobile Nursing and Care Settings in German-Speaking Countries: Delphi Study
Source: J Med Internet Res. 2025 Aug 13;27:e78193. doi: 10.2196/78193 (PMC12391839; doi:10.2196/78193)
Supplement: Multimedia Appendix 3 [file jmir_v27i1e78193_app3.docx]

Multimedia Appendix 3. Dimensions and key findings from the qualitative survey (phase 1) and thematic groups.

| Dimension | Key findings | Thematic group |
| --- | --- | --- |
| Throughput | - Low-threshold access to electronic health records (EHR) - Open and interoperable systems promote data exchange - Agile development processes enable continuous adaptation - Standardization of terminologies (e.g., SNOMED, LOINC) - Inclusion of various stakeholders in the development process - Funding requirements for stable digital infrastructures - Regulation of authorization systems within an EHR - Economic considerations to improve product quality - Handling of health data must be regulated to prevent misuse | - Implementation and evaluation - General necessity and national requirements - Digitalization in everyday healthcare - Standardization, classification systems, and terminologies - Research and development - Financing - Data authorization - Obligations and freedom of choice - Economic aspects - Data protection |
| Output | - Development of interdisciplinary digital applications - Cross-border data exchange is becoming increasingly important - Education and training for personnel are required - Implementation and integration of telemonitoring and related products into electronic health records - Addressing the potentials and risks of artificial intelligence (AI) | - Relevant functions of an electronic health record - Usability and support - International health data exchange - Training and interdisciplinary teamwork - Telemonitoring - Role of AI |
| Outcome | - Reducing administrative tasks to ease the burden on nursing staff - Improved flow of information and optimized workflows - Increased patient safety and reduced error rates - Enhanced cross-sectoral care and continuous patient support - Promotion of transparency and quality improvement in patient care - International data exchange fosters research and improves care solutions - Digitalization enhances data exchange in disaster and crisis situations | - Effects of interdisciplinary data exchange - Data exchange in disaster and crisis situations - Implementation and evaluation - Standardization, classification systems, and terminologies - Role of AI |
